# Bumblebee learning and memory is impaired by chronic exposure to a neonicotinoid pesticide

**DOI:** 10.1038/srep16508

**Published:** 2015-11-16

**Authors:** Dara A. Stanley, Karen E. Smith, Nigel E. Raine

**Affiliations:** 1 School of Biological Sciences, Royal Holloway University of London, Egham, Surrey, TW20 0EX, UK; 2 School of Environmental Sciences, University of Guelph, Guelph, Ontario, N1G 2W1, Canada

## Abstract

Bumblebees are exposed to pesticides applied for crop protection while foraging on treated plants, with increasing evidence suggesting that this sublethal exposure has implications for pollinator declines. The challenges of navigating and learning to manipulate many different flowers underline the critical role learning plays for the foraging success and survival of bees. We assessed the impacts of both acute and chronic exposure to field-realistic levels of a widely applied neonicotinoid insecticide, thiamethoxam, on bumblebee odour learning and memory. Although bees exposed to acute doses showed conditioned responses less frequently than controls, we found no difference in the number of individuals able to learn at field-realistic exposure levels. However, following chronic pesticide exposure, bees exposed to field-realistic levels learnt more slowly and their short-term memory was significantly impaired following exposure to 2.4 ppb pesticide. These results indicate that field-realistic pesticide exposure can have appreciable impacts on learning and memory, with potential implications for essential individual behaviour and colony fitness.

Bees are essential pollinators of many important agricultural crops and wild plants^1^, but declines in this group have been recorded worldwide^2^,^3^,^4^. There are many potential drivers of these declines including loss of habitat and disease, but one of the major factors is the intensification of farming and associated increased usage of agrochemicals^5^,^6^,^7^. Neonicotinoids are a major class of widely used pesticides that act systemically when applied to the seeds of crops, travelling through the plant tissues to target sucking pests^8^. Non-target organisms, such as bees, can be exposed to these pesticides (or insecticides) via residues found in pollen and nectar which can persist long after application^9^. Crops treated with these pesticides, such as oilseed rape (canola), can flower for several weeks^10^; therefore individual bees have the potential to be exposed to them for a substantial period during their foraging career resulting in longer-term, chronic exposure. Although neonicotinoids should not have lethal effects on bees at these trace levels, there is growing evidence of sublethal effects including impaired foraging ability^11^,^12^,^13^, reduced reproductive output^14^,^15^ and decreased navigation performance^16^,^17^.

Bees forage in the environment for pollen and nectar from flowers to feed themselves and their larvae. To collect these essential food resources bees display a diverse and sophisticated set of behaviours that rely heavily on learning and memory. These include navigating through a complex environment to find flower patches and return to their nest site, learning which cues (such as colour, scent and texture) are reliable predictors of floral reward from a diverse array of flower species, acquiring and fine-tuning the complex motor skills required to efficiently extract pollen and nectar from a variety of flower species, and learning to recognise and avoid potential predators^18^.

Neonicotinoids bind to and activate nicotinic acetylcholine receptors (nAChRs)^19^ affecting normal patterns of information transmission through the nervous system. They can cause neuronal inactivation in the mushroom bodies of the honeybee brain^20^, structures strongly associated with cognition, learning and memory in insects^21^. It is therefore possible that an underlying cause for the sublethal behavioural effects reported to date, such as reduced pollen foraging efficiency^11^,^12^, is that the learning and memory abilities of worker bees are impaired by neonicotinoid exposure.

Bumblebees (*Bombus* spp.) are a key group of social bees that perform essential pollination services for a wide range of commercially important crops and wild plant species^10^,^22^,^23^,^24^. However, to date studies investigating possible neonicotinoid impacts on learning and memory have been performed exclusively on honeybees^25^,^26^,^27^, a bee species with a rather atypical life history and ecology. Given the striking differences in biology between bumblebees and honeybees, the sensitivity of individual bees and colonies could be markedly different in these taxa^28^,^29^. A successful bumblebee colony, starting from a single foundress queen in spring, may produce a few hundred workers and persist for a number of months in an annual life cycle. In contrast, honeybee colonies are perennial; during winter the colony may contain only a few thousand individuals while strong summer colonies can reach populations of over 50,000 individuals. While all honeybee workers will become foragers for a relatively short period at the end of their life (average 7 days;^30^), bumblebee workers that forage may do this for their entire lifetime (which can be over 70 days;^31^). Furthermore bumblebees appear to be both more sensitive to the same levels of exposure, and metabolise neonicotinoids more slowly than honeybees^32^. Taken together these factors suggest that individual bumblebee workers are potentially at greater risk of sublethal effects than honeybees for a given level of pesticide exposure^33^.

The aim of this study was to test whether exposure to field-realistic levels of the neonicotinoid thiamethoxam has impacts on the learning and memory performance of the bumblebee (*Bombus terrestris*). We tested the olfactory learning performance of individual bees using the proboscis extension reflex (PER) conditioning paradigm. This is a commonly used method for assessing olfactory learning performance in honeybees^34^ and has been used in a number of studies investigating pesticide effects in this species^25^,^26^,^27^,^35^. This experimental paradigm has historically been challenging to use with bumblebees and has only recently been optimised successfully for studies of learning in *B. terrestris*^36^,^37^.

Bees were exposed to the pesticide thiamethoxam as it is the most widely used neonicotinoid seed dressing on oilseed rape in the UK, used on over 300,000 hectares of this crop in 2012^38^. It is also one of three neonicotinoids currently under a moratorium for use in the EU on crops attractive to bees, scheduled for reviewed in 2015 (Regulation (EU) No 485/2013). We used thiamethoxam exposure levels within the range that bees could encounter when foraging for pollen and nectar on treated crops (2.4 & 10 ppb:^39^,^40^,^41^) in two separate experiments designed to mimic different field-realistic scenarios. In the first experiment we exposed individual bumblebees to a small volume of pesticide treated sugar water to mimic the exposure they would get while foraging on a small number of oilseed rape flowers (acute exposure during a single foraging trip). In the second experiment we exposed entire bumblebee colonies to pesticide treated sugar water for a period of 24 days to mimic the situation of a colony foraging exclusively on a treated crop for its entire flowering period^42^ (chronic exposure). We then tested learning ability, and subsequent memory retention, using PER conditioning where individuals were trained to associate an odour as a predictor of reward. This allowed us to test trainability (whether bees learnt the association between odour and reward, or not, over the training period), learning level (how frequently bees showed they had learned the association between odour and reward by extending their proboscis to the trained odour alone), learning speed (the first odour presentation during the training period to which a bee first showed the learned association by proboscis extension), and memory (whether bees remembered the association between odour and reward after a 3-hour break following conditioning).

## Results

### Experiment 1: Acute exposure

**Figure 1:**
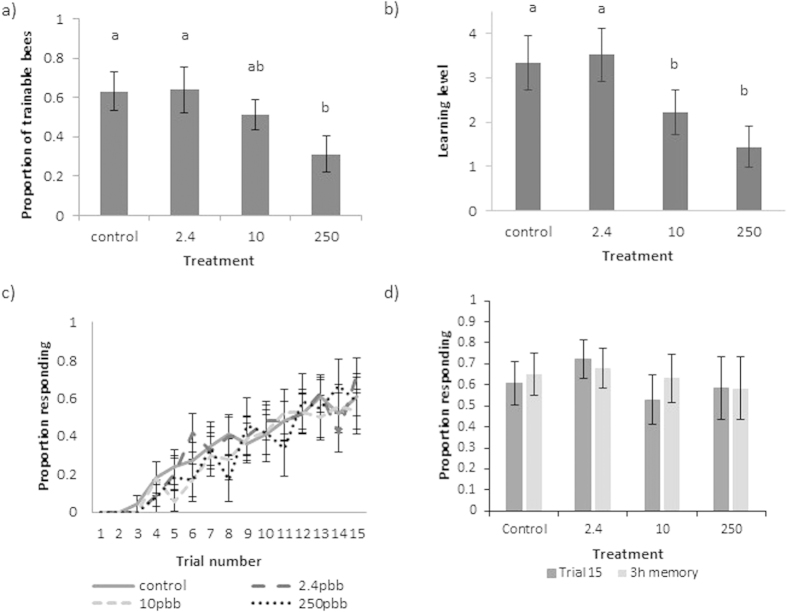
Results from experiment 1: acute exposure. (**a**) The mean proportion of bees in each acute treatment group that were trainable (trainability). (**b**) The mean number of conditioned responses of all acutely exposed bees per treatment group (learning level). (**c**) Acquisition curves showing the mean proportion of acutely exposed bees responding with a proboscis extension to the conditioned odour prior to reward over 15 conditioning trials. (**d**) Memory recall of the conditioned association (illustrated by mean proportion of bees that showed the conditioned response to the presented odour on trial 15 (dark grey bars) and 3 hours after the learning task in the memory test (light grey bars)) from trainable bees). Letters indicate significantly different pairwise comparisons from post-hoc tests (p < 0.05), and error bars indicate SE.

The olfactory learning performance of 171 individual bees from 6 colonies was tested. Bees that extended their proboscis to fewer than 5 odour presentations when their antennae were touched were classed as unresponsive and excluded from further analyses (n = 29: Table S1), resulting in an average of 35.5 bees tested per treatment (34 in control, 37 in 250 ppb, 36 in 10 ppb and 35 in 2.4 ppb treatment groups). Pesticide treatment affected both the trainability and learning level of bees (Fig. 1, Table 1a). More bees were trainable to the conditioned odour in the control and 2.4 ppb groups compared to the 250 ppb treatment group (Fig. 1a). Control bees also displayed a higher learning level than those from both the 10 ppb and 250 ppb treatment groups (Fig. 1b, Table 1a). While there was no significant difference between control and 2.4 ppb groups, post-hoc comparisons revealed that 2.4 ppb treated bees showed a higher learning level than both the 250 ppb (Tukey, Z value = 5.694, p < 0.0001) and 10 ppb (Tukey, Z value = 3.479, p = 0.0028) groups (Fig. 1b). We found no difference in worker body size across treatment groups (linear mixed effects model, F_3,164_ = 0.28, p = 0.8396), although there was a significant effect of body size in some models as larger bees showed a higher overall learning level (Table 1).

The learning ability of trainable bees (n = 78 bees in total: 23 bees in control, 24 in 2.4 ppb, 19 in 10 ppb, and 12 in 250 ppb treatments) was not affected by treatment (Fig. 1). Control bees neither learned the task quicker (Table S2b), nor displayed the conditioned response more frequently (Fig. 1d), than the other treatment groups, with the average bee responding to the odour for the first time at trial 8 (Table S2, Electronic Supplementary Material (ESM)). Similarly, the performance of bees in the memory task was not significantly different after three hours compared to the end of the training period for any treatment group (compare dark and lighter grey columns in Fig. 1d, related samples Wilcoxon signed rank tests: 2.4 ppb p = 0.715; 10 ppb p = 0.180; 250 ppb p = 0.655; control p = 0.317), indicating there was no overall impact of acute pesticide exposure on memory performance.

### Experiment 2: Chronic exposure

**Figure 2:**
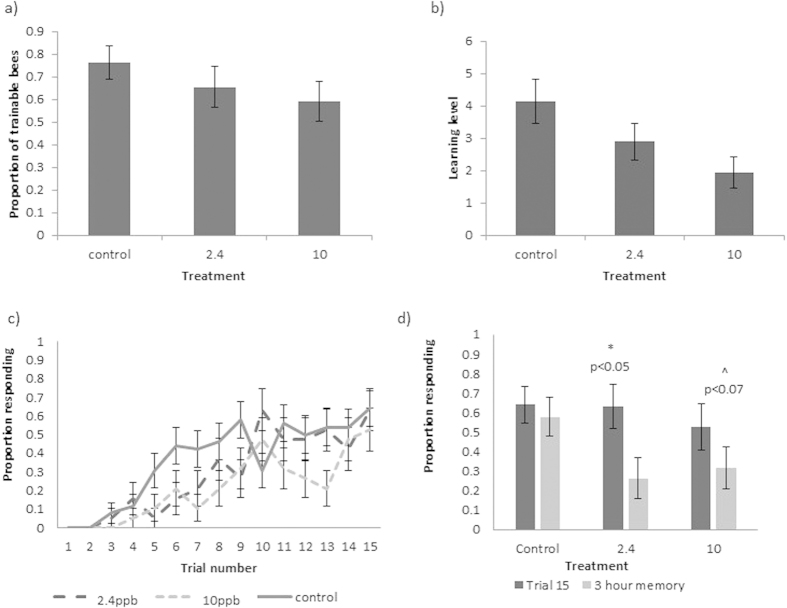
Results from experiment 2: chronic exposure. (**a**) The mean proportion of bees in each chronic treatment group that were trainable (trainability). (**b**) The mean number of conditioned responses of all chronically exposed bees per treatment group (learning level). (**c**) Acquisition curves showing the mean proportion of chronically exposed bees responding with a proboscis extension to the conditioned odour prior to reward over 15 conditioning trials. (**d**) Memory recall of the conditioned association (illustrated by mean proportion of bees that showed the conditioned response to the presented odour on trial 15 (dark grey bars) and 3 hours after the learning task in the memory test (light grey bars)) from trainable bees). Letters indicate significant differences (p < 0.05) and error bars show SE.

We tested the learning performance of 100 bees from 20 colonies (5 bees per colony), of which 5 unresponsive bees were removed from our analysis, resulting in 34 bees tested in control, 29 in 2.4 ppb and 32 in 10 ppb treatments (95 bees in total). We found no effect of pesticide exposure on either the number of bees that were trainable (Fig. 2a) or their learning level (Fig. 2b, Table S3). However, comparing only the performance of trainable bees (26 bees in control, 19 in 2.4 ppb and 19 in 10 ppb treatments: 64 bees in total), we found that control bees learnt the task faster than bees in both the 2.4 ppb (27% faster) and 10 ppb (38% faster) treatment groups (i.e. on average, the first response by control bees happened earlier in the experiment (mean = trial 6.9) than for pesticide treated bees, with average first responses at trial 8.7 for 2.4 ppb and 9.5 for 10 ppb (Table 2b), although final levels of task performance in terms of the proportion of bees responding to the conditioned odour and the learning level of these individuals was comparable across treatment groups after 15 trials (Fig. 2a, d, Table 2a)).

The 3-hour period between the end of conditioning and the memory test had no significant impact on the proportion of control bees displaying conditioned responses to the odour (Related samples Wilcoxon signed ranked test, p = 0.317; Fig. 2d). However, the proportion of bees exposed to 2.4 ppb pesticide that showed the conditioned response to the odour was significantly lower after the 3 hour break compared to the end of the trial period (Related samples Wilcoxon signed ranked test, p = 0.027), showing an impact of pesticide on memory. Although the proportion of bees in the 10 ppb exposure group responding to the odour stimulus after 3 hours was lower than at the end of the trial period, this difference was not significant at the 5% level (Related samples Wilcoxon signed ranked test, p = 0.066). There were no significant differences in worker body size across treatment groups (Linear mixed effects model, F_2,17_ = 2.83, p = 0.0869, although there was a trend for 10 ppb treated bees to be smaller).

## Discussion

This is the first study to investigate the effects of field-realistic exposure to a neonicotinoid pesticide on learning and memory in bumblebees. We investigated the effects on learning and memory performance of both acute (analogous to an individual bee visiting multiple flowers from a systemically treated crop during a single foraging bout) and chronic exposure (mimicking the levels a colony could experience when foraging on a neonicotinoid treated crop for 3–4 weeks) at field-realistic pesticide concentrations. We found minimal impacts of acute exposure on bumblebee learning and memory, although there was a significant reduction in the number of bees that were trainable at the highest dosage (250 ppb – positive control). However, while this shows a toxicity effect of acute exposure on trainability, this 250 ppb dose far exceeds the levels likely to be encountered in a field realistic scenario and so is not discussed further. In contrast, following chronic exposure at field-realistic levels bees were slower to learn the task, showed memory impairment after 3 hours, and there was also a trend for fewer bees to be trainable in the task with increasing thiamethoxam concentration (Table 3).

Reduced speeds of learning an association as a result of chronic neonicotinoid exposure could have significant knock-on effects for colony development in the wild, as intercolony variation in learning speed has been shown to correlate with foraging performance in the field^43^. We found that bees exposed to pesticide took 27% (2.4 ppb) to 38% (10 ppb) more trials to learn than controls. As such, colonies containing impaired learners could be more constrained in the floral resources they can collect and invest into colony growth and reproduction. Bees typically forage in an environment containing dozens of flower species that differ in colour, scent and morphology and also the quantity and quality of rewards they provide. Foraging bees need to learn to exploit the most rewarding floral sources that will change over time. Hence if a bee takes longer to learn floral cues as predictors of reward they may miss out on more profitable flowers^18^.

The additional finding that a bee’s 3 hour memory is significantly impaired following exposure to 2.4 ppb pesticide means that bees exposed to pesticide may have to spend additional time re-learning how to handle morphological complex flowers and/or the location of rewarding patches. Although bees from all treatment groups ended up reaching the same level of task performance at the end of the conditioning period in our study, the differences in rates of learning took place over a 3-hour test period. As bumblebee foraging bouts generally last less than 80 minutes^11^,^12^, pesticide impacts on both the speed of learning over a 3-hour (180 minute) test period (the duration of the PER conditioning period) coupled to impacts on memory performance (3 hours after conditioning ceased) have the ability to influence performance across multiple foraging bouts. Interestingly, we did not find significant impairment of memory recall comparing bees exposed to 10 ppb pesticide and control. This may have been due to a small sample size (although as p = 0.07 this comparison is approaching significance at the 5% level). Alternatively this may be indicative of hormesis^44^, where certain lower levels of pesticide exposure may actually stimulate bee brains causing a different outcome.

Previous studies have also shown that imidacloprid (another neonicotinoid) exposed bumblebees foraged less efficiently for pollen^11^,^12^,^13^. As pollen foraging is a more complex task to learn than nectar collection^45^, our results suggest these findings could be related to impairment of their ability to learn and/or remember salient cue-reward associations. Taken together with previous evidence of behavioural impairment to pollen foraging^11^,^12^,^13^ this provides another example of how slower learning could negatively affect the dynamics and efficiency of individual bee foraging, colony growth and development.

Our study adds to a growing body of evidence on the sublethal effects of pesticides on learning in honeybees, including studies reporting impairment^26^,^27^,^35^,^46^, no impact^47^ and facilitation of cognitive performance^25^,^48^ (although many of these previous studies report sublethal impacts following exposure to pesticide levels considerably above field realistic levels). Results from our chronic exposure experiment are similar to a honeybee study that showed impaired learning and memory when chronically exposed to imidacloprid for 4 days^46^. Interestingly, these effects were reversible if the honeybees were given 3 days to recover without ingesting additional pesticides. As bumblebees also show increased activity and a recovery in feeding rate associated with clearing the pesticide from their bodies after 48 hours^32^, it would be useful to investigate whether bumblebees also show signs of impacts on learning and memory becoming ameliorated when pesticide exposure ceases. However, because honeybees and bumblebees vary appreciably in aspects of their physiology, ecology, life history and ability to metabolise pesticides (honeybees can constantly metabolise imidacloprid (125 μg/L) under lab conditions ensuring a daily clearance of 100%^32^, although bumblebees start to accumulate the pesticide after 24 hours exposure with a clearance rate of only 68%) this could also mean they are differentially affected by similar pesticide exposure. Unlike honeybees that can communicate information about profitable reward sites through the waggle dance, bumblebees primarily have to learn which flowers are rewarding by individual experience. Although bumblebees can communicate odour information^49^,^50^ to nest mates, they are unable to communicate about specific locations, meaning foragers need to explore to find rewarding flower patches for themselves. This suggests that the ability of individuals to learn foraging related cues is potentially more important for individual bumblebees than honeybees, and any (pesticide) impacts on cognitive ability may therefore have more severe consequences for bumblebees.

In our study we aimed to be as ecologically relevant as possible by using levels of pesticide reported from field situations^39^,^40^,^51^,^52^,^53^ and exposure regimes that bees could easily encounter when foraging. Treated crops, such as oilseed rape, can be visited by large numbers of bumblebee colonies^54^, but our exposure scenario is probably conservative as test colonies only received pesticide treated nectar whilst under field conditions bees are exposed to both contaminated nectar and pollen. However, having our bees restrained between pesticide treatment and learning tests (1 hour acute, 20 hours chronic) is somewhat un-natural, as in a field situation bees would have the opportunity to fly potentially increasing their ability to metabolise the pesticide. Alternatively bees could either consume a much higher volume of pesticide during a foraging bout in the field, or continue to forage before they are able to metabolise the pesticide, therefore the effects on learning and memory we report here could be magnified in the field. As highlighted in previous work, bees can be exposed to multiple pesticides in field realistic conditions^11^, which may have different impacts on learning performance^25^,^46^. It would be interesting to mimic a similar situation in bumblebees, for example combining thiamethoxam with a spray applied pesticide (e.g. lambda-cyhalothrin) and investigating whether potential effects on learning and memory are different.

We have shown that field-realistic concentrations of thiamethoxam have minimal effects on bumblebee learning and memory following acute exposure, but workers were slower to learn and showed impaired 3-hour memory after 3–4 week, chronic exposure. This adds to the body of evidence showing sublethal effects of neonicotinoids on bumblebees and is the first to show learning and memory deficits in bumblebees after chronic exposure, such as if the colony lived close to a seed treated field of oilseed rape. Deficits in learning and/or memory following chronic exposure have implications for many tasks essential for successful bumblebee reproduction (including foraging, navigation, brood care), and could be a sublethal impact of pesticides not recorded in other studies. Our findings indicate the pressing need to assess potential chronic and sublethal impacts of pesticides on a variety of bee species during the regulatory pesticide risk assessment process. Key differences between results obtained on different bee taxa indicate that results from honeybees cannot simply be extrapolated to bumblebees or solitary bees when making policy decisions. Balancing the risks of using neonicotinoids as a systemic seed treatment to control economically important pests against unintended impacts on non-target beneficial insects (including essential pollinators) requires a rapid increase in our understanding of the impacts of these pesticides on bees and other organisms.

## Methods

### Pesticide exposure

A stock pesticide solution was made by dissolving 10 mg thiamethoxam (Sigma Aldrich) in 100 ml Acetone. Aliquots of stock solution were added to 40% sucrose (v/v) to create the following pesticide concentrations: 250 parts per billion (ppb; acute experiment only), 10 ppb and 2.4 ppb. The highest concentration (250 ppb) was chosen as a positive control for the acute experiment as this level, far above those to which bees are likely to be exposed in the field, would be expected to have an effect (based on exposure levels causing no bumblebee mortality after 48 hours of exposure (Baron *et al.*, unpublished data), and approximately 42% of no observable effect level (NOEL) honeybee LD_50_^55^). Concentrations of 2.4 and 10 ppb were chosen to be within field relevant ranges: 2.4 ppb was informed by thiamethoxam levels detected in nectar pots of *B. terrestris* colonies foraging in agricultural areas^40^ and 10 ppb is at upper end of the range likely to be found in pollen and nectar of treated crops^39^,^51^,^52^,^53^ and wild flowers^15^,^56^,^57^. Control solutions were made by repeating the dilution process above using an aliquot of 10 ppb acetone containing no pesticide.

### Experiment 1: Acute exposure

We obtained six bumblebee (*Bombus terrestris audax*) colonies from Biobest (Westerlo, Belgium) in March 2014, each containing a queen and an average of 66 workers. Each colony was transferred to a bipartite wooden nest-box and connected to a flight arena (60 × 100 × 35 cm). Untreated honeybee collected pollen (frozen and defrosted prior to feeding) was provided directly into the nest-box every two days and 40% untreated sucrose solution (v/v) was provided *ad libitum* from a gravity feeder in the flight arena. As these were commercially reared colonies that were delivered directly from the rearing facility to our laboratory we assumed they had no prior exposure to pesticide.

**Figure 3:**
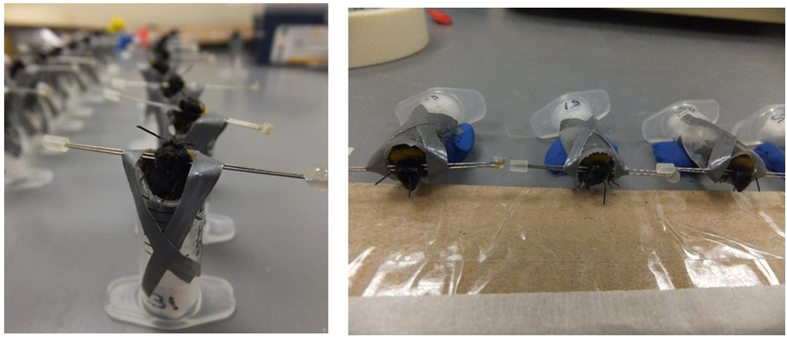
Harnessed bees prior to PER testing (a) and in horizontal positions after feeding in the acute experiment (b). Bee harnesses were held in place with a piece of modelling clay (blue plasticine) and their head set above a piece of plastic covered cardboard (used for easy disposal the next day to prevent cross treatment contamination) on which sucrose solution was presented (photos by DAS). For additional details about the harnessing methodology see Smith & Raine^37^.

Each week colonies were randomly assigned to two groups to have their learning ability tested on separate consecutive days. Over four weeks an average of 37 foraging bees (range 17–42) from each colony were captured from the gravity feeder in the flight arena and harnessed (Fig. 3 37). Bees were fed with 40% sucrose solution 2 hours after harnessing, and placed in a horizontal position in a dark room overnight (Fig. 3). The following morning, bees were randomly assigned within colony to be fed 10 μl of control or 250 ppb, 10 ppb or 2.4 ppb thiamethoxam solution. The solution was pipetted onto plastic covered cardboard (Fig. 3b). The bee’s antennae were touched with untreated 40% sucrose solution to elicit proboscis extension for them to drink the droplet. Once the bees had consumed the entire droplet they were placed in an upright position and the learning task began one hour later (this time delay was chosen to ensure bees had started to metabolise the pesticide, and was estimated for bumblebees from reported rates of honeybees^55^).

Learning was assessed using the proboscis extension reflex (PER) paradigm: a type of classical conditioning in which bees learn to associate a floral odour (conditioned stimulus) with a sucrose solution (unconditioned stimulus) reward^58^. Bees were conditioned using a natural odour blend (lemon essential oil, Calmer solutions) paired with a 50% sucrose solution (v/v) reward (a higher concentration than provided prior to the experiment to stimulate bee participation in the task), and they had to learn to associate this odour as a predictor of reward. Each bee was trained individually in an odour extraction hood every time it was exposed to the odour. An odour tube (approx. 3 cm away) pointed towards the bee (containing 1 μl of the essential oil odour on a piece of filter paper) delivered a precise stimulus to the bee. A programmable logic controller (PLC) computer controlled the volume of air, flow rate and duration of stimulus presentation to each bee. Odour tubes were changed every 20–30 uses to maximise consistency of odour strength. Bees were first presented with clean air for 5 s and then the air containing the odour for 10 s (15 seconds of airflow from the PLC in total); therefore there was consistent air flow across the bee to which the conditioning odour was added. The reward (0.8 μl of 50% sucrose solution) was presented to the bee 6 s into the odour stimulus by touching their antenna using a Gilmont syringe to elicit proboscis extension, and then allowing them to consume the droplet. The odour was presented to the bee 15 times with an inter-trial interval (ITI) of 12 minutes. Once a bee learnt the association between the odour stimulus and reward they extended their proboscis as soon as the odour was presented (prior to reward delivery). For each odour presentation we recorded whether the bee responded to the conditioned odour prior to reward delivery or not giving us a binary (yes or no) response. Individual memory was assessed by recording the response of each individual to a single presentation of the odour three hours after PER conditioning had finished. Three hours was chosen due to the limitations of working with bumblebees rather than honeybees. Pilot work showed that a large proportion of bumblebees died or were un-responsive when left in harnesses for 24 hours. Therefore we chose a time period for memory recall within the range used previously (i.e. 2 or 5 hours) for the only study that has assessed this parameter in bumblebees^59^. Following the experiment the body size (thorax width) of all the bees was measured with digital callipers across the widest part of the thorax.

### Experiment 2: Chronic exposure

Twenty-one *B. terrestris audax* colonies were obtained from Biobest in April 2014, each containing a queen and approximately 70 workers. Colonies were weighed on arrival to estimate size, ranked in decreasing mass order, then placed into 7 groups of three colonies. On day 1 the first group of colonies (containing the 3 heaviest colonies) were assigned at random to three treatment groups (10 ppb thiamethoxam, 2.4 ppb thiamethoxam and control). On day 2 the second group (containing the 4^th^–6^th^ heaviest colonies) were allocated, until day 7 when the final three colonies were allocated. This process ensured that each colony was exposed to their treatment solution for the same time period due to subsequent sequential PER testing. Colonies were fed untreated pollen (as above) every 2–3 days and their treatment sucrose solution *ad libitum* in a gravity feeder inserted at the base of the commercial colony box. During the treatment period all colonies were given some foraging experience (each colony was connected to a flight arena for 48 hours in the laboratory and then allowed 1–2 hours exposure to apple trees in an outdoor flight cage which had not been treated with pesticide). Other than this very brief period of exposure to an alternative, untreated nectar source bees would have consumed the treated sucrose and therefore exposure is assumed to be relatively uniform.

Colonies were tested using PER conditioning after being exposed to treatments for an average of 24 days (range 22–26). This time period was chosen to mimic a situation in which a colony may forage on oilseed rape during its entire flowering period. Five colonies were sampled on each day, and testing continued for 4 days until 20 colonies had been tested (one 2.4 ppb colony was excluded from testing as it produced large numbers of males earlier in the colony cycle and so was at a later reproductive stage than the others). Six workers that exited each colony box were caught, and harnessed as explained above (Fig. 3). Two hours after harnessing, bees were fed untreated 40% sucrose solution and then left in a dark room overnight. The following morning, responsiveness was tested by touching their antenna with a droplet of untreated 50% sucrose solution. Those bees responding with a proboscis extension were fed a small droplet of this untreated solution before PER testing began 15 minutes later as described above (resulting in control, n = 34; 2.4 ppb, n = 29; 10 ppb, n = 32).

### Analysis

A number of variables to describe learning performance were extracted from the PER results from both experiments including: (1) trainability - whether bees learnt the association between odour and reward (or not) over the 15 presentations (binary response), (2) learning level - total number of learnt responses (proboscis extensions in anticipation of reward), (3) learning speed - trial number when the first learnt response occurred. Differences among treatment groups were tested for each of these response variables using generalized linear mixed models (GLMMs) in lme4 package^60^ in R version 3.1.0^61^, assuming binomial distributions for binary, and poisson distributions for count, data. We included both treatment and bee body size as predictor variables in all models. In addition, colony membership was included as a random effect in all models (in both acute and chronic experiments) and week of testing was included as a random effect in acute experiment models. We also tested for differences in worker body size among treatment groups using a linear mixed effects model with colony as a random factor in the nlme package^62^. All models were validated by assessing normal Q-Q plots and residual versus fitted data plots. To investigate differences between individual treatment groups, pairwise post-hoc comparisons were performed using the multcomp package^63^. To assess pesticide impacts on memory after 3 hours, we compared conditioned responses on the 15^th^ trial with those 3 hours later by calculating the proportion of conditioned responses from each colony and then compared among colonies for each treatment group using a pairwise repeated samples Wilcoxon test. Trainability and learning level were analysed for all bees, and then we subsequently analysed learning level and learning speed including only those bees that were trainable.

## Additional Information

**How to cite this article**: Stanley, D. A. *et al.* Bumblebee learning and memory is impaired by chronic exposure to a neonicotinoid pesticide. *Sci. Rep.* **5**, 16508; doi: 10.1038/srep16508 (2015).

## Supplementary Material

Supplementary Information

## Figures and Tables

**Table 1:** Generalized linear mixed models for (a) the binary trainability response variable and (b) the learning level variable in the acute experiment (experiment 1) using all exposed bees that took part in the task (n = 142 bees).

	Fixed effects	Parameterestimate	SE	Z value	P value
(a) *Trainability*	Intercept (Control)	−8.53	3.34	−2.56	0.011
	Treatment (250 ppb)	−1.63	0.53	−3.05	**0.002**
	Treatment (10 ppb)	−0.71	0.51	−1.40	0.164
	Treatment (2.4 ppb)	0.07	0.53	0.13	0.895
	Bee size	1.86	0.67	2.78	**0.005**
(b) *Learning level*	Intercept (Control)	−1.87	1.06	−1.77	0.077
	Treatment (250 ppb)	−0.79	0.17	−4.70	**<0.0001**
	Treatment (10 ppb)	−0.38	0.15	−2.63	**0.009**
	Treatment (2.4 ppb)	0.07	0.13	0.53	0.599
	Bee size	0.55	0.20	2.70	**0.007**

Parameter estimates are calculated with reference to the control group. Colony and week of PER testing were included as random effects. Significant p values (p < 0.05) are highlighted in bold.

**Table 2:** Generalized linear mixed models for the (a) learning level responses variable, (b) learning speed response variable for chronically exposed bees that were trainable in the learning task (n = 64 bees: experiment 2).

	Fixed effects	Parameterestimate	SE	Z value	P value
(a) *Learning level*	Intercept (Control)	−0.63	1.24	−0.51	0.61
	Treatment (10 ppb)	−0.42	0.25	−1.70	0.09
	Treatment (2.4 ppb)	−0.19	0.24	−0.79	0.43
	Bee size	0.43	0.24	1.80	0.07
(b) *Learning speed*	Intercept (Control)	1.73	0.84	2.06	0.039
	Treatment (10 ppb)	0.33	0.11	3.00	**0.003**
	Treatment (2.4 ppb)	0.24	0.11	2.20	**0.027**
	Bee size	0.04	0.16	0.24	0.81

Parameter estimates are calculated with reference to the control treatment. Colony is included as a random effect. Significant p values are highlighted in bold.

**Table 3:** Summary table of main results for both acute (experiment 1) and chronic exposure (experiment 2).

Response variable	Acute exposure (Expt 1)	Chronic exposure (Expt 2)
Trainability	Negative effect at 250 ppb	No effect
Learning level (all bees)	Negative effect at 10 and 250 ppb	No effect
Learning level (only bees that were trainable)	No effect	No effect
Learning speed	No effect	Negative effect at 2.4 and 10 ppb
Memory	No effect	Negative effect at 2.4 ppb

“No effect” indicates that there was no significant difference (p > 0.05) between any pesticide exposure levels (2.4, 10 and 250 ppb for acute experiment, 2.4 and 10 ppb for chronic experiment) and the control treatment. This table is for illustration purposes only as experiment 1 and 2 are not directly comparable due to differences in methodologies.
