# Supplementary material for: Bumblebee learning and memory is impaired by chronic exposure to a neonicotinoid pesticide
Source: Sci Rep. 2015 Nov 16;5:16508. doi: 10.1038/srep16508 (PMC4644970; doi:10.1038/srep16508)
Supplement: Supplementary Information [file srep16508-s1.doc]

**Bumblebee learning and memory is impaired by chronic exposure to a neonicotinoid pesticide**

**Electronic Supplementary Material**

Dara A. Stanley^1*^, Karen E. Smith^1*^, Nigel E. Raine^1,2^

^1^School of Biological Sciences, Royal Holloway University of London, Egham, Surrey, TW20 0EX, UK; ^2^School of Environmental Sciences, University of Guelph, Guelph, Ontario, N1G 2W1, Canada.

^*^These authors contributed equally to the work

Email: [darastanley@gmail.com](mailto:darastanley@gmail.com)

**Experiment 1: Acute exposure**

***Unresponsive bees***

Twenty nine (*ca*. 17%) of the 171 acutely exposed bees that entered PER conditioning did not extend their proboscis when their antennae were touched on more than 5/15 of the conditioning trials. These bees were excluded from the analysis and classed as insufficiently responsive to participate. We found no difference in the number of unresponsive bees among treatment groups when each was compared to the control in the acute experiment (Table S1).

**Table S1.** Generalized linear mixed effects model for unresponsive bees after acute exposure (experiment 1). Parameter estimates were calculated with reference to the control group. Colony was included as a random effect (n = 29 bees).

| **Fixed effects** | **Parameter estimate** | **SE** | **Z value** | **P value** |
| --- | --- | --- | --- | --- |
| Intercept (Control) | 1.792 | 3.276 | 0.547 | 0.584 |
| Treatment (250 ppb) | 0.495 | 0.579 | 0.855 | 0.393 |
| Treatment (10 ppb) | 0.645 | 0.607 | 0.059 | 0.953 |
| Treatment (2.4 ppb) | 0.031 | 0.530 | 1.063 | 0.288 |
| Bee size | -0.092 | 0.640 | -0.142 | 0.887 |

a)

b)

**Table S2.** Generalized linear mixed effects models for (a) the learning level and (b) the learning speed of bees that were trainable in the acute experiment (experiment 1). Parameter estimates were calculated with reference to the control group. Colony and week were included as random effects (n = 78).

|  | **Fixed effects** | **Parameter estimate** | **SE** | **Z value** | **P value** |
| --- | --- | --- | --- | --- | --- |
| 1. *Learning level* | Intercept (Control) | 0.08 | 0.95 | 0.08 | 0.93 |
|  | Treatment (250 ppb) | -0.13 | 0.17 | -0.75 | 0.45 |
|  | Treatment (10 ppb) | -0.18 | 0.15 | -1.21 | 0.23 |
|  | Treatment (2.4 ppb) | 0.04 | 0.13 | 0.31 | 0.76 |
|  | Bee size | 0.29 | 0.19 | 1.49 | 0.14 |
|  |  |  |  |  |  |
| 1. *Learning speed* | Intercept (Control) | 1.76 | 0.65 | 2.7 | 0.01 |
|  | Treatment (250 ppb) | 0.05 | 0.12 | 0.4 | 0.69 |
|  | Treatment (10 ppb) | -0.07 | 0.11 | -0.6 | 0.54 |
|  | Treatment (2.4 ppb) | -0.03 | 0.10 | -0.30 | 0.77 |
|  | Bee size | 0.07 | 0.13 | 0.54 | 0.59 |

**Experiment 2: Chronic exposure**

***Unresponsive bees***

Five of the 100 (5%) chronically exposed bees that entered the PER conditioning did not extend their proboscis when their antennae were touched on more than 5/15 of the conditioning trials. These bees were excluded from the experiment and classed as insufficiently responsive to participate.

We found a trend indicating that a lower proportion of bees were able to learn the task (trainability) as thiamethoxam concentration increased, although this trend was not statistically significant (see Table S3a for model output, Figure 2i). In addition, we found that bees showed a greater number of correct responses in the control group compared to the 2.4 ppb and 10 ppb treatments, however this different was not significant at the 5% level (see Table S3b for model output, Figure 2ii). We found a significant effect of worker body size, with larger bees giving a greater number of correct responses (for model output see Table S3b, p = 0.008).

**Table S3.** Generalized linear mixed effects models for the (a) trainability response variable and (b) learning level response variable for chronically exposed bees (experiment 2). Parameter estimates were calculated with reference to the control treatment. Colony was included as a random effect. Significant values are highlighted in bold, n =95.

|  | **Fixed effects** | **Parameter estimate** | **SE** | **Z value** | **P value** |
| --- | --- | --- | --- | --- | --- |
| 1. *Trainability* | Intercept (Control) | -4.239 | 4.55 | -0.932 | 0.351 |
|  | Treatment (10ppb) | -0.693 | 0.551 | -1.258 | 0.209 |
|  | Treatment (2.4ppb) | -0.592 | 0.570 | -1.039 | 0.299 |
|  | Bee size | 1.083 | 0.910 | 1.190 | 0.234 |
|  |  |  |  |  |  |
| 1. *Learning level* | Intercept | -2.038 | 1.261 | -1.616 | 0.106 |
|  | Treatment (10ppb) | -0.743 | 0.386 | -1.924 | 0.054 |
|  | Treatment (2.4ppb) | -0.359 | 0.389 | -0.923 | 0.356 |
|  | Bee size | 0.642 | 0.243 | 2.638 | **0.008** |
